# Compensatory Health Beliefs on Breastfeeding Varying by Breastfeeding Status; A Scale Development

**DOI:** 10.3390/ijerph17165759

**Published:** 2020-08-09

**Authors:** Efrat Neter, Levana Bagants

**Affiliations:** Department of Behavioural Sciences, Ruppin Academic Center, Emeq Hefer 4025000, Israel; levanapi@gmail.com

**Keywords:** Breastfeeding, compensatory health beliefs, dissonance, attitude, counselling

## Abstract

Aims: To examine whether compensatory health beliefs (CHB) on breastfeeding vary as a function of breastfeeding status among mothers of infants. Methods: Participants included 773 women aged 18 and older (M = 32.8) who gave birth in the last two years; 445 were breastfeeding exclusively, 165 were breastfeeding partially, and 163 were not breastfeeding. They responded to a survey posted on social media sites’ closed groups that focused on post-natal issues. Design was cross-sectional, with CHB as the outcome variable (14 items) and demographics and feeding status as the explanatory variables. Results: The internal reliability of the CHB scale was α = 0.87. There was a statistically significant difference in the level of CHB between non-breastfeeding women, breastfeeding women, and women who combined breastfeeding with infant formula, so that non- breastfeeding women had the highest level of CHB. There was no significant difference in CHB by either birth experience or demographic characteristics. Conclusion: This study extended CHB to breastfeeding, documenting the minimization of the disadvantages of not breastfeeding by non-breastfeeding women, attempting to neutralize or reduce the cognitive dissonance between non-nursing and optimal infant care. Possible uses of the scale for counselling were suggested, both in the prenatal and post-natal period, proactively bringing forward and addressing ambivalence towards breastfeeding.

## 1. Introduction

Though breastfeeding is documented as providing many health benefits for both new-borns and mothers and the WHO recommendations are to exclusively breastfeed babies at least until 6 months old, many women do not breastfeed exclusively or at all. These women may use compensatory health beliefs (CHB) to neutralize or reduce the cognitive dissonance between non-nursing and optimal infant care. Capturing this ambivalence towards breastfeeding could assist in counselling, either prenatally or at the postnatal period. The present work extends work on compensatory health beliefs in other domains (smoking, eating, exercising) into breastfeeding; it examines beliefs on breastfeeding of mothers to new-borns by their breastfeeding status (exclusive, partial, none) and birth experiences.

Breastfeeding is endorsed and encouraged internationally [1,2,3]. Exclusive breastfeeding, defined as an infant receiving “only breastmilk, no other liquids or solids” from birth to six months, is advocated because of its advantages both to the infant and the mother. 

The advantages to the infant are both immediate and beyond the period of breastfeeding: Breastmilk is nutritionally superior to commercial formulas, it protects from infectious diseases or malnutrition caused by contaminated water or over-dilution of breastmilk substitutes [3], and its long-term effects include lower risk of obesity and diabetes, higher intelligence, reduced malocclusion, and reduced asthma [3,4,5]. In terms of maternal health, breastfeeding was found to be associated with a more rapid return of the uterus to its pre-pregnant state, a more rapid return to pre-pregnancy weight or weight loss, and it affects glucose and lipid metabolism which may have implication for later development of chronic conditions [6]. Indeed, there is growing evidence that extended breastfeeding is associated with reduced risk of premenopausal breast-, ovarian-, and endometrial cancers, type II diabetes, and postpartum depression [3,4,7]. 

Breastfeeding is espoused as the “normative” mode of infant feeding [2] and a mainstay of global child health. Indeed, breastfeeding is considered by some “as a legal right of the child and the promotion of breastfeeding as a legal obligation of countries that ratified the UN Convention on the Rights of the Child” [7]. A global strategy ensued, comprised of setting national breastfeeding policies and coordinators, setting breastfeeding as a core measure of hospital quality, ensuring that all countries enact legislation making the workplace environment friendly for breastfeeding mothers and monitoring the marketing of breastmilk substitutes [8,9,10].

Though this global strategy on breastfeeding is manifested in national programs, organizational policies (hospitals, worplaces), public opinion [11,12], and even social media [13,14,15], in many countries exclusive breastfeeding often falls short of the national goals and recommended targets [4,7,16]. Though the determinants of breastfeeding are manifold and include of structural level (e.g., market context), settings level (e.g., workplace), and individual level components (e.g., infant’s temperament or maternal stress [17]), women who do not breastfeed are faced with the information, images and norms [12] that portray breastfeeding as ideal for babies and the perception that breastfeeding mothers as demonstrate greater devotion [7]. This discrepancy is also evident in Israel, a country with strong pro-natal policies and attitudes [18], yet with exclusive breastfeeding rate (54.8% at 6 months) which falls short of the goal [19]. 

The above-mentioned discrepancy between one’s behavior—not breastfeeding at all or not breastfeeding exclusively, on the one hand, and the medical information and social norms supporting breastfeeding and equating good and devoted motherhood with breastfeeding [12], on the other hand, creates a cognitive dissonance. One way of resolving the discrepancy or justifying the choice/behavior of not breastfeeding is by creating compensatory health beliefs (CHB), which neutralize or reduce the harmful effects of the choice/behavior. CHB are a self-defence strategy employed when people experience a mental conflict between cognitions and behaviors or among cognitions [20,21]. The best way to reduce this cognitive dissonance would be to act in accordance with the preferred behavior, e.g., to quit smoking or to maintain breastfeeding, but as these behaviors are difficult to implement, people opt for the next best option: Compensating for the negative effects of the habit (smoking, over eating) by engaging in another promoting heath behaviors such as exercising, or by generating CHB which minimize the negative effects of their behavior (e.g., “you have to die of something”) [22,23]. CHB allow people to maintain unhealthy behaviors because they ameliorate the negative feelings associated with these behavior and allow people to preserve their positive self-image. 

CHBs have been linked to a variety of health behaviors, for example eating, smoking [24], physical activity [25], alcohol consumption [26], and vaccination [27,28]. For example, compared to people who had high intentions to vaccinate, those with low intentions to vaccinate for influenza were more likely to believe that their healthy lifestyle protected them from influenza; furthermore, the adoption of such CHB was associated with decreased uptake of the flu shot [27]. CHBs have not been examined, as of yet, in the context of breastfeeding; consequently, existing CHBs scales address other behaviors (exercise, smoking, eating) and were deemed as unsatisfactory for breastfeeding. 

### The Present Study

In the present work, we extended CHB to breastfeeding. First, we developed a CHB scale specific to the breastfeeding context (see Table 1) and then examined the hypothesis that CHBs pertaining to breastfeeding (e.g., formula is good enough, bonding can be achieved in other ways, and suffering in breastfeeding is not worth the goal) form a gradient, so that they are the highest among non-breastfeeding mothers, followed by mothers who combine formula and breastfeeeding and the lowest among mothers who breastfeed exclusively. The reasoning is that the first two groups have to justify their “non-normative” behavior to themselves and to their surroundings. Additionally, CHBs were examined as a function of birth experience (infant and maternal complications), a stressor that may affect breastfeeding.

## 2. Methods

### 2.1. Participants 

A questionnaire was addressed to women over 18 years-old who had given birth in the last two years. It was completed by respondents who clicked on a link (*n* = 1040) and expressed their agreement to participate by ticking an “I agree” box. Complete data were available from 773 women (74.3% completion rate) aged 18 to 49 years (M = 32.86, SD = 4.52), 445 (57.6%) of them exclusively breastfeeding, 165 (21.3%) exclusively feeding with a formula and 163 (21.1%) partially breastfeeding. Full demographics are displayed in Table 2. 

### 2.2. Design and Procedure

The study design was cross-sectional. The outcome variable was compensatory health beliefs and the explanatory variables were feeding status and birth experience. 

A link to an online “survey on breastfeeding” was posted on 2018 in Hebrew-speaking social media sites closed groups (*n* = 6) that focused on post-natal issues (both Facebook and WhatsApp groups). Women read a Plain Language Statement about the aims of the study and its focus on their perceptions. They were provided with information on the researchers and ways of contacting them and gave explicit consent (ticking a box in an online survey). The completion time of the survey was approximately 20 min duration. Participants did not provide personally identifying information and data was further anonymized by removing IP address, longitude and latitude data in the stored final dataset.

The study was approved by an Internal Review Board of Ruppin Academic Center (Review Number: 2019-43_L/bs). 

## 3. Measures

### 3.1. Breastfeeding Status

This was determined by the response to the item: “How do you feed your baby?” with the following response options: Exclusive breastfeeding, formula or a combination of formula and breastfeeding (=partial breastfeeding). Women who reported on breastfeeding were asked how long they had been breastfeeding.

### 3.2. Birth Experience

This measure was assessed by two items, asking respondents whether there were maternal and/or infant complications during birth. Response options were yes/no, and if a complication occurred, women were asked to provide details. 

### 3.3. Compensatory Health Beliefs on Breastfeeding (CHB-BF)

This was assessed using a scale developed by the authors (see Table 1). The scale included 15 items; responses were given using a 5-point Likert scale, ranging from “strongly disagree” to “strongly agree”. Items were scored such that a high score indicated high compensatory beliefs. Items are displayed in Table 1. A total mean score of 15 beliefs was computed (α = 0.87, M = 2.67, SD = 0.63).

### 3.4. Demographic Information

Characteristics of age, education, social-economic status, and marital status were recorded.

### 3.5. Data Analysis: Scale Development

Items were generated using findings from previous work [16,29,30] and interviews with women (*n* = 3). A pilot version of the scale was administered to 6 respondents using cognitive interviewing [31,32]: Respondents were asked to “think aloud” as they completed the scale and the researcher occasionally probed when they hesitated or indicated a misunderstanding. Occasionally, after completion, the researcher asked several probing follow-up questions related to items which took the respondents longer to answer. In this way, insight was gained into the readability and clarity of the items and items were altered accordingly. The scale was thus revised twice. 

### 3.6. Scale Analysis

The psychometric quality of the CHB-BF was tested using item analysis, reliability estimation and exploratory principle component factor analysis (principal components method of extraction, varimax rotation) to explore potential dimensions of CHB on breastfeeding. A total mean score of CHB-BF was computed and basic descriptive statistics were computed both for the total sample and by feeding modality. Subsequently, the hypothesis and research question on differences in CHB-BF by feeding modality and (adverse) birth experience were tested using planned comparisons. Lastly, a post-hoc examination of the association between CHB-BF and demographic attributes was carried out by computing a Pearson correlation or a χ2. The analyses were performed using SPSS 23 [33]. 

## 4. Results

### 4.1. Participants’ Characteristics

Participants’ age ranged from 18–49 and the interquartile range was in the 30s (30–36 years-old). Most of the sample had attained tertiary-level education and reported on an income of average and above. The vast majority were married or in cohabitation and the average age of the infant on whose feeding they reported on his/her feeding was on average 1-year old, with an interquartile range of 5-17.50 months. See Table 2 for full details.

### 4.2. Reliability and Structure of the CHB-BF Scale

We performed reliability analysis and exploratory factor analysis (EFA) with principal component analysis and varimax rotation using the following criteria: (1) Eigenvalue greater than 1, (2) items loading on the same factor (≥0.30), (3) no cross-loading at similar values, (4) Cattell’s scree test, and (5) interpretability of factors. The internal consistency of the scale was α = 0.87. Table 1 lists the items, item–total correlations from the reliability analysis, their factor loadings from the EFA after rotation and factor affiliation (a, b, or c). As can be seen, most items have an item–total correlation exceeding the threshold of 0.40; two items (#5, #14) have values hovering on the threshold (0.38, 0.39) and one item (#6) has an unsatisfactory low item–total correlation of *r* = 0.27. Removal of item #6 did not change the reliability (α = 0.87) nor did the further removal of the items #5 and #14 (still, α = 0.87). 

Three factors with eigenvalues greater than 1 emerged from the analyses; the eigenvalues for these factors were 5.16, 1.38, and 1.09, explaining 36.83%, 9.83%, and 7.80% of the variance. The factors explained accumulatively 54.46% of the variance. The factor loadings displayed in Table 1 indicate satisfactory factor loadings. The first factor was comprised of most items (#1, #2, # 4, #5, #7, #8, #9, #10, #11, #12, #13, and #15) and relates to health and illness, adverse effects of breastfeeding and infant-mother interaction. The second factor was comprised of only two items (#3 and #14) and can be considered as a challenge to the normative discourse on breastfeeding (on health and bonding). It should be noted that one of the items (#3) had also a high loading also on the first factor, albeit smaller. The third factor was comprised of only one item (#6) which also had low item–total correlation; this item had dissatisfactory loading on the first two factors. Even when a two-dimension solution was constrained, the item did not load satisfactorily (<0.40) on any of the two dimensions. The correlations between the first two factors (*r* = 0.47) demonstrated satisfactory discriminant validity. 

### 4.3. Comparing the CHB of the Breastfeeding Groups 

**Figure 1:**
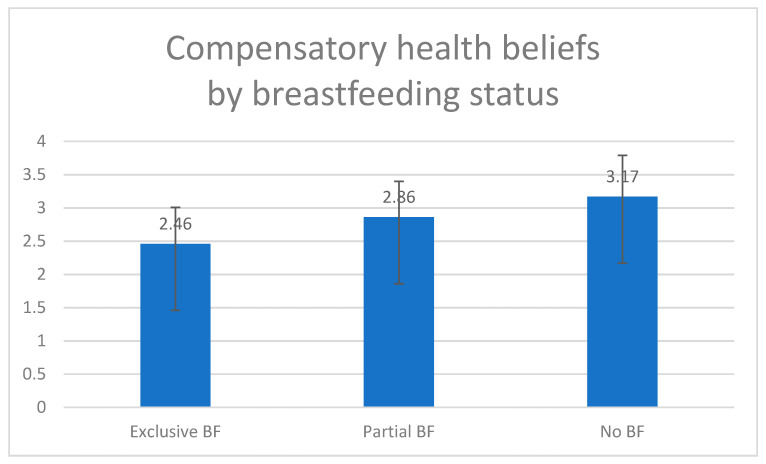
Compensatory health beliefs by breastfeeding status (Mean, SD). Note: BF = breastfeeding. *F* (2,770) = 98.88, *p* < 0.001, *η^2^* = 0.20.

An analysis of variance yielded a significant difference between the groups, *F* (2,770) = 98.88, *p* < 0.001, *η^2^* = 0.20. The groups’ means are displayed in Figure 1. Two planned comparisons were carried out on CHB: (1) Comparing breastfeeding women (exclusively or in combination with a formula) to non-breastfeeding women and (2) comparing women who exclusively breastfed to those who breastfed in combination with a formula. Breastfeeding mothers had a significantly lower CHB score than did non-breastfeeding mothers, *t* (770) = 7.79, *p* < 0.001, and also mothers who exclusively breastfed had a significantly lower CHB score than did those who breastfed in combination with a formula, *t* (770) = 7.46, *p* < 0.001.

Finally, comparisons of CHB as a function of birth experiences and demographic characteristics were carried out. CHB did not differ for women who experienced either maternal complications *t* (771) = 0.49, *p* = 0.63 or infant complications *t* (771) = 0.31, *p* = 0.75. Similarly, CHB was not associated with the age of the women, their education or reported income (*p*’s > 0.05); however, there was a small significant negative association between the age of the infant and CHB, so that as the infant grew, the mothers held less CHB (*r* = −0.08, *p* = 0.042).

## 5. Discussion

### 5.1. Summary

This work extended the study of compensatory health beliefs into the context of breastfeeding. A new scale (CHB-BF) was developed and found to be psychometrically sound. As part of the construct validation of the scale, it was found that non-breastfeeding women held more CHB than did breastfeeding women, and that women who combined breastfeeding with formula held more CHB than did women who breastfed exclusively. No association was found between CHB-BF and either the demographic attributes or the birth experiences of the women.

### 5.2. Comparison with Prior Work 

The current work corroborated findings that have been documented mostly in qualitative literature of conflicting or ambivalent attitudes towards breastfeeding [30]. It shows women as having both positive and negative feelings about breastfeeding and diverges from an “overly romanticized discourse on maternal identity” ([29], p. 244 ). As feminist theorists reported [29], the experience of breastfeeding is diverse. For some women, it can be viewed as connected to their lifestyle and values and feel harmonious and sensual, yet for other women breastfeeding can be painful, disrupted, and disconnected with personal values, such as autonomy, independence, and control. Being part of the latter group raises contradictions between one’s experience and the “pro-breastfeeding discourse” ([29], p. 241). The emergence of a separate factor in CHB-BF, which focused on challenging the dominant views regarding the benefits of breastfeeding in terms of both health and bonding, reverberates qualitative findings on challenging the fundamental assumption of “breast is best” [34]. It may indicate that women who did not have a positive experience breastfeeding were concomitantly struggling to maintain a positive view of themselves as good mothers [34], defending their choices by justifying their deviation from the prescribed behavior [34]. Furthermore, the finding that the CHBs were not associated with demographic attributes may indicate that these beliefs are pervasive. 

Our findings echo other findings in the literature on CHB. Though no previous work on CHB addressed breastfeeding, studies on other health behaviors provide ample empirical evidence of holding CHB as a means of ameliorating cognitive discomfort in various situations. For example, smoking-specific CHBs were found to be significantly negatively related to the intention to stop smoking [24]; alcohol-specific CHB were associated with an increase in alcohol consumption [26]; and post-exercise beliefs licensing the consumption of unhealthy snacks were higher among people driven by control motivation (rather than by autonomy) [35]. In the context of breastfeeding, our findings indicate that women who do not breastfeed (or do not do so exclusively) hold more CHBs, suggesting an attempt to resolve the contradiction between how they view themselves as mothers and the normative social portrayal of breastfeeding, which was probably partially internalized. The CHBs represent their defiance of the dominant views. 

### 5.3. Limitations and Strengths

The developed scale proved psychometrically adequate: the internal reliability was high, each dimension explained more than 5% variance and accumulatively the dimensions explained 54% of the total variance, considered satisfactory. Still, the scale could be improved. Specifically, dropping the one item (#6) in the third dimension is advisable, as its item–total correlations was not satisfactory and neither were its loadings on any of the other two dimensions when a two-factor solution was forced. 

The current work is innovative in its application of CHB in the context of breastfeeding. Yet, as an initial first step, it harbors several limitations. First, the study is cross-sectional. Clearly, the sequence between CHB-BF and the behavior itself, or what preceded this behavior, was not examined. Nor was the possibility of compensatory behavior (in relation to non-breastfeeding) [24] examined; for example, whether non-breastfeeding mothers spend more time with their babies. Related antecedents or moderators, examined in other studies in more oft-researched areas, were also not addressed: For example, whether self-efficacy in breastfeeding [20,26] is associated with CHB. Lastly, the data collection method—the online convenience survey—resulted in a non-representative sample of Israeli birth-giving women, specifically this sample was more affluent and more educated than the population of women of this age group in the country. A selection bias, possibly leading to non-representativeness, could not be estimated, as the number of women potentially exposed to the survey could not be determined: the online groups where the survey was posted were already long-established (i.e., existed for several years), with the records indicating the cumulative number of members over the years but no indication of membership during the period of the current study. 

The study exhibits several strengths. First, the sample was relatively large (*n* = 773). Second, our sample adequately represented breastfeeding in Israel: exclusive breastfeeding was reported among 57.6% of our sample, which is close to the figure reported (54.8%) in an epidemiological survey [19]. Third, it generated robust engagement among responders: there were many comments and requests for the study’s results (which were later posted in the social media sites where the study was advertised). Lastly, the new scale offers opportunities in giving a voice and space to non-breastfeeding women and in opening up opportunities to better understand the experience of breastfeeding and develop novel counselling on breastfeeding. The negative experience of some women in breastfeeding needs to be acknowledged, thus possibly addressing some of the hindrances and issues.

## 6. Conclusions

The findings clearly give a voice to women who did not connect to breastfeeding and are either not breastfeeding at all or combine formula and breastfeeding. The scale affords raising diverse issues in breastfeeding counselling—from its view as “personalized medicine” [4] to possible adverse effects (pain, sore nipples) and mother-infant bonding. One of the affordances of using the scale is that it questions, challenges, and explores the dominant views. This is one of the first quantitative studies that attest to the ambivalence and complexity of women’s views on breastfeeding.

Future work could extend the findings. First, the scale could be improved (removing one item, improving phrasing) and tested in other cultures; it would be of interest if similar findings would emerge (or not) in less pro-natal societies. Theoretically, future work could examine the motivations for breastfeeding (for example, control vs. autonomy) and whether each motivation is associated with CHB [35]. Self-efficacy is another construct that could unravel whether the ambivalent attitudes identified are the consequence of low self-efficacy or are more associated with issues of autonomy and selfhood. Ambivalence towards breastfeeding itself could be further explored. Specifically, examining the dimensions of ambivalence (valence, polarity, and complexity) using Kaplan’s split semantic differential [36], could identify the dimension/s most prevalent in women’s ambivalence towards breastfeeding. Lastly, a longitudinal design in future work could establish the sequence between CHC-BF, self-efficacy, and breastfeeding behavior. 

At the policy level, the scale could be utilized in the context of counselling, both in pre- and post-natal periods. In the pre-natal period, the scale could be used by health care practitioners as a trigger to provide information and proactively bring up issues of adverse effects as well as autonomy and suspended selfhood. Encountering these issues early on, by including not only the themes of baby’s health and bonding [30], can provide women with the time to process also the potential difficulties and search for ways of coping. The healthcare practitioner could also provide information on resources that address the specific difficulties the counselee feels she may have. This could serve as a preparation that acknowledges the challenge of breastfeeding. In the post-natal period, the scale could be used in breastfeeding counselling or women’s health professionals either to address difficulties in breastfeeding or to support the decision the woman made, acknowledging that besides structural or societal predictors, there are also individual-level factors associated with breastfeeding. 

## Figures and Tables

**Table 1:** Corrected item–total correlation and factor structure of the 15-item compensatory health beliefs on breastfeeding (CHB-BF) scale.

Items	Item-Total Correlation	Loading on Factor (a, b, c)
1. We all grew up on formula and turned out OK	0.59	0.68 a
2. The formula has identical nutritional components to those of breastmilk	0.47	0.56 a
3. A baby can be healthy in spite of not being breast fed.	0.47	0.55 b
4. I know babies fed on formula who were less ill than those on breastmilk.	0.42	0.49 a
5. A mother not breastfeeding is less likely to undergo postpartum depression	*0.38*	0.47 a
6. When breastfeeding is not frustrating me, my baby is less frustrated	*0.27*	0.73 c
7. Time devoted to breastfeeding can be channeled to self-fulfillment	0.54	0.63 a
8. Not breastfeeding allows for balance in life	0.66	0.74 a
9. Breastfeeding is enslaving and dependent, and my sanity is more important.	0.67	0.77 a
10. Not breastfeeding alleviates engorgement and sore nipples	0.58	0.54 a
11. I don’t think spending time with your baby should be misery, even if it’s for a good cause as breastfeeding	0.56	0.47 a
12. Not breastfeeding allows for more quality time of games with the baby	0.65	0.74 a
13. Not breastfeeding means more availability and dedication to the baby’s needs	0.66	0.77 a
14. A baby may be completely bonded with the mother in spite of not being breast fed.	*0.39*	0.61 b
15. If engorgement and sore nipples make me exasperated towards myself and the baby, it’s better not to breastfeed.	0.57	0.64 a

Note: Item-total correlations <0.40 are displayed in italic font. a—health and illness, adverse effects of breastfeeding and infant-mother interaction; b—challenge to normative discourse on breastfeeding (on health and bonding); c—baby’s response.

**Table 2:** Sample characteristics (*N* = 773).

Variable	*N* (%)	Mean (SD)
Age, in years		
Range; Interquartile Range	18–49; 30–36	
Mean (SD)		32.86 (4.52)
Education	*N* (%)	
Secondary	65 (8.4)	
Post-secondary	114 (14.7)	
Tertiary	594 (76.9)	
Income	*N* (%)	
Average and above	593 (76.7)	
Marital Status	*N* (%)	
Married/cohabitating	757 (97.9)	
Divorced/widowed/Separated	6 (0.8)	
Single	10 (1.3)	
Infant’s age in months		
Mean (SD); Interquartile Range		12.15 (9.11); 5–17.50

## Data Availability

The datasets used and/or analyzed during the current study are available from the corresponding author upon request.
